# Development and validation of a self-care scale for older adults undergoing hip fracture surgery: the HFS-SC

**DOI:** 10.1186/s12912-022-00982-3

**Published:** 2022-07-22

**Authors:** Eun-Jeong Jeon, Kyeong-Yae Sohng, Hye-Ah Yeom

**Affiliations:** College of Nursing, The Catholic University of Korea, Seoul, Republic of Korea

**Keywords:** Hip fractures, Aged, Self care, Instrumentation

## Abstract

**Background:**

The ability to take care of oneself after hip fracture surgery is important for older adults. Various scales have been developed for evaluation of this ability, but a scale specifically focusing on hip fracture has not been developed. The aim of this study was to develop and validate a scale (Hip Fracture Surgery Self-Care Scale, HFS-SC) to evaluate self-care for older adults undergoing hip fracture surgery.

**Methods:**

The scale was developed according to the guidelines by DeVellis. Initial items were derived from a literature review and individual interviews with 11 older adults who underwent hip fracture surgery. To confirm the suitability of the questions, a preliminary survey was conducted on 25 older adults. Psychometric testing was performed on 300 older adults 65 years old or over living at home after surgery for hip fracture. Psychometric properties of the scale were examined by content validity, construct validity, concurrent validity, internal consistency reliability, and test-retest reliability.

**Results:**

Exploratory factor analysis and confirmatory factor analysis demonstrated that the 18-item scale comprised five factors (functional independence, symptom recognition and management, positive mental health, participation and support in social activities, and a safe environment). The results of EFA showed that the factor loadings ranging from 0.51 to 0.87. The results of CFA were χ2=375.83, χ2/*df*=2.14, RMSEA 0.07, SRMR 0.05, GFI 0.88, TLI 0.91, and CFI 0.92 for the 18-item scale. The reliabilities of the scale were 0.91 for Cronbach’s alpha and 0.82 for test-retest reliability.

**Conclusions:**

The HFS-SC has acceptable validity and reliability and is expected to be useful for evaluating the levels of self-care for older adults undergoing hip fracture surgery and adjusting at the post-fracture period at the community or out-patient department.

**Supplementary Information:**

The online version contains supplementary material available at 10.1186/s12912-022-00982-3.

## Background

A hip fracture (HF), which refers to a break in the continuity of the femur at the site of its articulation with the pelvis [1], is a serious clinical event associated with high morbidity and mortality rates in older adults [2]. HF commonly occurs in individuals aged $\ge$ 80 years [3]. The incidence of HF has nearly doubled in the past decade in South Korea, and the mortality rate within one year from fracture diagnosis has increased to 20% [4]. The incidence rate of HF in South Korea was approximately 376 per 10,000 individuals in 2016 and continues to steadily increase by 4% every year [5]. Usually, surgery is recommended in most patients to minimize complications of HF [6]. Restricted functional performance and difficulties with activities of daily living (ADLs) negatively affect quality of life in older adults who undergo surgery for treatment of HF [7–9]. Bone regeneration does not usually occur at sites of HF. Patients invariably require prolonged postoperative hospitalization, and usually older adults do not recover their full pre-fracture functional performance at the time of discharge [8]. Furthermore, >10% of older adults with HF do not return home after discharge, which contributes to a significant social and economic burden on long-term care facilities [10, 11]. Therefore, it is important to develop strategies to enable patients’ return to their pre-fracture status and ADLs through appropriate self-care.

Self-care is defined as an activity performed by an individual to maintain life, health, and well-being [12]. Self-care consists of the following main concepts: universal, developmental, and health-deviated self-care. Universal self-care includes activities that are common to all humans and those that are necessary to maintain daily life. Developmental self-care includes activities required during developmental process in various situations. Health-deviated self-care refer to activities associated with disease states, diagnosis, and treatment [12]. The self-care theory proposed by Orem [13] focuses on enhancement of the self-care ability of individuals with restricted mobility and is regarded as adequate for individuals with HF [13]. Based on Orem’s self-care theory, scales used to measure self-care, such as the Exercise of Self-Care Agency (ESCA) [14], Perceived Self-Care Agency Questionnaire (PSCAQ) [15], and Self-care Capability Scale (LSCS) [16], which were developed between the late 1990s and the 2000s are useful to assess self-care in the general population [17]. However, these scales to not include HF-specific items that reflect characteristics of older adults with HF, which highlights the need to establish a self-care scale that may be useful in older adults with HF.

Older adults who undergo HF surgery require high levels of self-care for functional recovery [18]. Although self-care is important to maintain patients’ quality of life, a scale that measures self-care in older adults who undergo HF remains unavailable. General evaluation scales, such as the Activities of Daily Living (ADL) [19, 20], Independence in the Activities of Daily Living (IADL) [21, 22], and EuroQol-5Dimensions 5Levels (EQ-5D-5L) scales [23] are often used to evaluate the functional level in older adults who undergo HF surgery. Although these scales are widely used, the items included in the evaluation are not specific to self-care for HF surgery. For example, ADL items are associated with the physical aspects of function, whereas the instrumental activities of daily living scale focuses on cognitive function such as phone use or shopping. Although the EQ-5D-5L contains multidimensional indicators, it includes a wide range of items and is not developed for a specific disease or age group. This scale is limited for measurements of the environmental and social aspects of self-care in older adults who undergo HF surgery. A scale that includes specific attributes such as emotional aspects (anxiety, depression, or fear of falls), social aspects (social activities, relationships, and support), environmental aspects (safe living environment), and cognitive and physical aspects (awareness of one’s health status and self-care performance) is best suited to evaluate self-care in older adults who undergo HF surgery.

Self-care assessment plays an important role in the evaluation of the recovery stage and establishment of treatment and educational strategies to improve quality of life in older adults who undergo HF surgery. In this study, we aimed to develop a scale that can comprehensively measure self-care levels in older adults who undergo HF surgery, primarily by focusing on their transition to daily living at home, and examined the validity and reliability of the scale.

## Methods

**Fig. 1:**
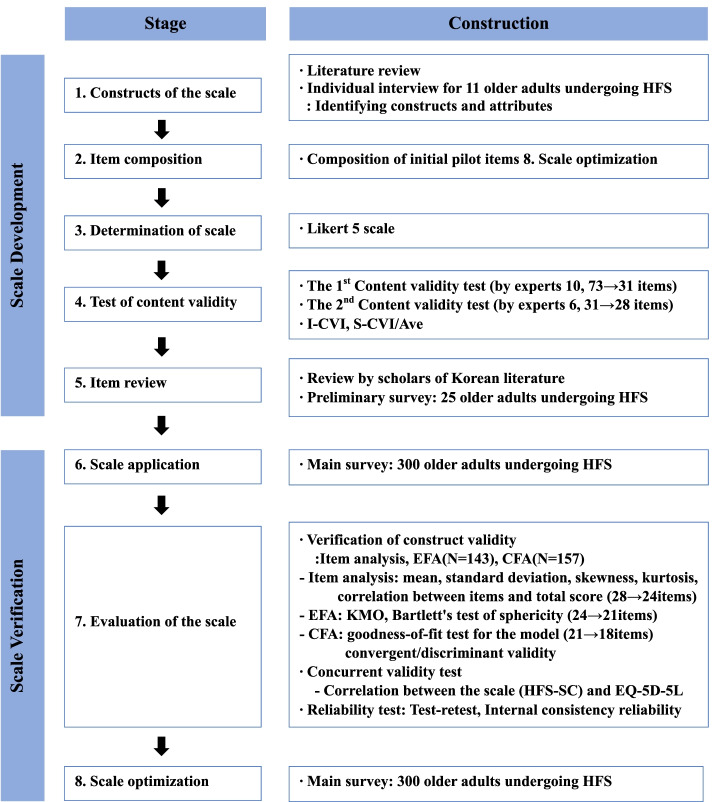
Process of scale development

This study examined the reliability and validity of the Self-Care Scale for Older Adults undergoing Hip Fracture Surgery (The HFS-SC). All methods were carried out following the ethical guidelines of the latest Helsinki declaration. The scale was developed based on the guidelines by DeVellis [24], which include scale development stage and scale examination stage. In the scale development stage, the constituent factors and properties of the self-care concept of older adults in hip fracture surgery were identified, and preliminary questions were constructed through a literature review and patient interviews. After content validity was tested by experts, a preliminary survey of the items was conducted with a sample of older adults who underwent HF surgery. In the scale examination stage, a main survey was conducted by applying the HFS-SC scale to older adults who underwent HF surgery. Final items were confirmed through construct validity, concurrent validity, and reliability tests (Fig. 1).

### Scale development process

#### Stage 1: Constructs of the scale

To analyze the concept of self-care in older adults in HF surgery, an integrated literature review method suggested by Whittemore and Knafl [25] was used. Using various search engine databases, the literature on self-care models, self-care concept analysis, self-care scales, and HF published up to August 31, 2020 were reviewed.

To specify the concept of self-care for older adults with HF surgery derived from the literature review, 11 subjects were individually interviewed from November to December, 2020 using a content analysis method. The validity and reliability of the data were secured through credibility, fittingness, auditability, and confirmability as suggested by Sandelowski [26]. Examples of the main questions for the interviews include the following: What do you think self-care is for HF patients? What efforts are you making to take care of yourself after HF surgery? After HF surgery, what were you most worried about in your daily life living at home?

#### Stage 2: Item composition

Based on the literature review and individual interviews, a total of 73 preliminary questions on the HFS-SC scale were derived.

#### Stage 3: Determination of scale

A 5-point (1 = *strongly disagree*; 2= *disagree*; 3= *neutral*; 4= *agree*; 5= *strongly agree*) Likert scale was used to measure the HFS-SC scale.

#### Stage 4: Test of content validity

The first content validity test was conducted by 10 experts, including 3 orthopedic surgeons, 2 nursing professors, and 5 nurses with more than 10 years of nursing experience in the orthopedic ward. Based on the results of the first content validity, the second content validity test was performed by 6 experts who participated in the first test. An Item-level content validity index (I-CVI) higher than 0.78 [27] and Scale-level content validity index/average (S-CVI/Ave) higher than 0.90 [28] were used as the item selection criteria.

#### *Stage 5: I*tem review and preliminary survey

Since the HFS-SC scale is aimed at older adults, some of the items were modified after consulting with a scholar with a major in the Korean language to check whether the grammar or overall sentence flow is appropriate and ensure that subjects can easily read and understand the items. Data for the preliminary survey were collected from 25 older adults in January 2021. After completion of the questionnaire, data were collected in a sealed envelope. Each item was evaluated from the standpoint of both understandability and clarity. The degree of understanding of each item was measured on a 4-point scale ranging from 1 (*very easy to understand*) to 4 (*very difficult to understand*). Items with high frequency of responses with 3 or 4 points or with an opinion requesting correction were revised accordingly. The clarity of each item was measured on a 5-point scale ranging from 1 (*very difficult to understand*) to 5 (*very easy to understand*).

#### Stage 6: Scale application

A large-sample survey was conducted to verify the validity and reliability of the HFS-SC. The inclusion criteria for study participants included those who underwent HF surgery at two hospitals in Gyeonggi-do, Korea within 2 months to 1 year, those who were able to walk before HF and had no abnormality in balance ability (including older adults using assistive device), those who are over 65 years old and who can communicate and respond to questionnaires, and those who understood the purpose of this study and agreed to participate. Exclusion criteria for study participants included those who were bed-bound, or those who were not able to make verbal communication due to stroke, dementia, or psychiatric disorders. The sample size was set to 300, as at least 100 subjects are needed for exploratory factor analysis [29] and at least 150 samples for confirmatory factor analysis [30] anticipating a dropout rate of 20% [31]. Study participants were requested to fill out the questionnaire voluntarily. The researcher, who was the author and the data collector, clarified the meanings of the items if participants required help, and was not involved in the completion of the survey questionnaire. After completion of the questionnaire, it was collected in a sealed envelope, and a gift certificate worth $5 was provided as compensation for study participation.

#### Stage 7: Evaluation of the scale

Construct validity was examined by item analysis, Exploratory factor analysis (EFA), and Confirmatory factor analysis (CFA). For item analysis, the normality of the items was confirmed by Skewness, Kurtosis, and Mardia coefficient. The corrected item-total correlation coefficient was conducted to confirm the relevance of each item to the scale, and only items with a correlation coefficient value of 0.3 or higher were selected for the scale [32, 33].

For factor analysis, the study participants were randomly divided into two groups using the random sample cases method using SPSS version 22.0. The first group was used for EFA (*n*=143), which was used to extract the factors from the scale. We conducted EFA through a five-step process [34]. Step 1 involved assessment of the data suitability for factor analysis using Kaiser-Mayer-Olkin (KMO) measure of sampling adequacy and Bartlett’s test of sphericity. Step 2 was the selection of factor extraction method. Of factor extraction methods (i.e., principal component analysis, principal axis factoring, maximum likelihood, alpha factoring, etc), we used Principal Component Analysis (PCA) as it is a common factor extraction method that can be applied to the case when no priori factor model exists [34]. Step 3 was the process of determining the number of factors. We used an Eigenvalue of 1.0 or higher and a Scree plot as the criteria to assist in concluding the number of factors. Step 4 included the selection of the rotation method, which was the orthogonal varimax rotation for this study. The items were selected based on factor loading higher than 0.5 [32], communality higher than 0.5, and accumulative variance higher than 60% [29]. Step 5 was the identification of a theme for each factor to assess variables attributable to the factor.

The other group was used for CFA (*n=*157). CFA was analyzed through the structural equation model estimated using Maximum likelihood estimator (MLE). The values of Root mean square error of approximation (RMSEA), Standardized root mean square residual (SRMR) and Goodness of fit index (GFI), Turker-lewis index (TLI), Comparative fit index (CFI) were included as the model fit indices. Convergent validity of subdomains was examined by confirming that the standardized factor loading was higher than 0.5, the Critical ratio (C.R) was higher than 1.96, the Average variance extracted (AVE) value was higher than 0.5, and the Construct reliability (CR) was higher than 0.7 [35]. Discriminant validity was evaluated by confirming that the square root of the variance extraction index of the model was larger than the correlation coefficient of each factor [36, 37].

Concurrent validity was examined through correlation analysis between the HFS-SC scale and the EQ-5D-5L. The EQ-5D-5L is a health-related quality of life measurement scale that consists of five domains (mobility, self-care, usual activities, pain/discomfort, anxiety/ depression). The EQ-5D-5L was used as a criterion scale because its subdomains contain the attributes of self-care and that there is no scale measuring self-care of older adults with HF. The EQ-5D-5L score is calculated by applying a weight to each area that is predicted by the estimation formula applied to calculate a value between the maximum value of 0.83 and the minimum value -0.07 [38].

Reliability of the HFS-SC was evaluated by Intra-class correlation coefficient (ICC), test-retest reliability, and internal consistency reliability [37].

## Results

### Scale development

#### Stage 1: Constructs of the scale

For the literature review, a total of 9,969 studies were retrieved from the search engines. Of the studies, 4,730 duplicate papers were excluded, resulting in a remaining 5,239 studies. After excluding 4,476 papers that were not related to this study, 763 papers were selected. Then, 731 articles were excluded based on the selection criteria. Of the remaining 32 papers, 3 were systematic review studies, which already had quality evaluation, and 29 papers were evaluated using the critical by Joanna Briggs Institute [39]. As a result, 13 studies with methodological flaws were excluded, resulting in the final 19 studies for review.

A review of the 19 studies showed that essential attributes of the domain of universal self-care were maintaining proper nutritional status, balance between rest and activity, assistance-independence balance, participation and support in social activities, improvement of the residential environment, and overcoming fear of falling. Important attributes of the domain of developmental self-care were self-efficacy, improvement of resilience, and stress management. In the domain of health-deviated self-care, the essential attributes derived from the literature review were health status recognition, problem-solving skills, knowledge acquisition, rehabilitation implementation, daily life change adaptation, and pain/discomfort management. A total of 14 factors and 52 priori items were developed from the literature review.

For patient interviews, 11 older adults of varying age, gender, education level, family type, religion, and time after operation were recruited. Through individual interviews, the domain of spiritual support was derived as an attribute of self-care after HF surgery and was thus added into the item pool. As a result, a total of 15 factors and 45 priori items were derived and validated from the individual interviews. Literature review and patient interviews were conducted simultaneously, resulting in 52 items from the literature review and 45 items from the individual interviews.

#### Stage 2: Item composition

Based on the literature review and patient interviews, similarities and differences among the final factors and priori items were identified through a conceptual reasoning process, which resulted in 7 factors with 73 preliminary items: 4 items for maintaining a normal nutritional status [7, 18], 17 items for effort in rehabilitation [8, 14, 40–44], 13 items for symptom recognition and management [7, 11, 43–46], 11 items for a safe environment [10, 11, 40, 44, 47, 48], 11 items for participation in and support for social activities [7, 10, 44, 49], 13 items for positive mental health, and 4 items for spiritual support [7, 11, 14, 40, 44, 45, 49, 50].

#### Stage 3: Determination of the scale

A 5-point Likert scale was used to measure the levels of SC in each item, ranging from 1 (*strongly disagree*) to 5 *(strongly agree*).

#### Stage 4: Test of content validity

For the primary content validity, only items with an I-CVI of 0.78 or higher were selected. If the meanings of the items were duplicated based on the expert opinions, they were revised by deletion, correction, and integration. Out of 73 preliminary questions, 11 questions were revised, and 42 questions were deleted, resulting in a total of 31 preliminary questions. In the process of examining S-CVI, 3 items were deleted, and 7 items were revised, leading to 28 items. The secondary content analysis test showed that the I-CVI of all items was 0.78 or higher, ranging from 0.83 to 1. The S-CVI/AVE was 0.95, which met the criterion value of 0.90 or higher, thus securing the content validity of the scale.

#### Stage 5: Item review

After the linguistic adequacy of the items was reviewed by a scholar with major in Korean literacy, 3 items were revised. The preliminary survey containing 28 questions was conducted on 25 older adults who underwent HF surgery. The average survey time was 19.63 min. The overall clarity score of the questions based on a 5-point scale was 3.85 $\pm$ .92, and the overall understandability of the questions based on a 4-point scale was 3.43 $\pm$ .78.

Scale verification

#### Stage 6: Scale application

Table 1 shows the general characteristics of the subjects. The average age of the participants was 83.25 years old, and 58.33% of the individuals were female. Regarding the educational level, 44.67% of the participants were uneducated. The majority of subjects lived with family. Approximately half of the participants were not employed, and 65.33% were religious. In functional state, 216 participants reported they were able to walk independently without the use of assistive device, whereas 86 individuals reported using cane or walker for walking. Regarding the type of surgery, 49.33% had total hip arthroplasty. The overall characteristics were homogeneous, as there were no significant differences in general characteristics between EFA (*n=*143) and CFA (*n=*157) groups (Table 1).

**Table 1:** General characteristics of participants for main survey (*N=*300)

Characteristics	Total(*n=*300)	EFA(*n=*143)	CFA(*n=*157)	Χ^2^ or t	*p*
	n(%) or M $\pm$ SD	n(%) or M $\pm$ SD	n(%) or M $\pm$ SD		
Age(year)					
(Range 65~89)	83.25 $\pm$ 5.36	82.97 $\pm$ 4.93	83.54 $\pm$ 5.78	.81	.54
Sex					
Male	125(41.67)	59(41.26)	66(42.04)	.01	.97
Female	175(58.33)	84(58.74)	91(57.96)		
Education level					
Uneducated	134(44.67)	65(45.45)	69(43.95)	3.84	.65
Elementary	85(28.33)	41(28.67)	44(28.03)		
Middle	37(12.33)	17(11.89)	20(12.73)		
High	44(14.67)	20(13.99)	24(15.29)		
Family type					
Spouse	126(42.00)	59(41.26)	67(42.68)	2.62	.51
Offspring	51(17.00)	22(15.38)	29(18.47)		
Spouse+ offspring	37(12.33)	21(14.69)	16(10.19)		
Solitary	86(28.67)	41(28.67)	45(28.66)		
Work					
Part-time	83(27.67)	37(25.87)	46(29.30)	.76	.48
Full-time	49(16.33)	24(16.78)	25(15.92)		
None	168(56.00)	82(57.34)	86(54.78)		
Religion					
Protestant	83(27.67)	40(27.97)	43(27.39)	1.24	.92
Catholic	47(15.66)	19(13.29)	28(17.83)		
Buddhism	66(22.00)	31(21.68)	35(22.29)		
None	104(34.67)	53(37.06)	51(32.48)		
Functional status					
Walking independently	216(72.00)	102(71.33)	114(72.61)	.07	.63
Walking with assistive device^a^	84(29.00)	41(28.67)	43(27.39)		
Type of HFS					
THR	148(49.33)	68(47.55)	80(50.96)	1.06	.75
BHA	82(27.33)	40(27.97)	42(26.75)		
PFNA	47(15.67)	23(16.08)	24(15.29)		
ORIF	23(7.67)	12(8.39)	11(7.00)		
Period of HFS(months)	9.17 $\pm$ 1.53	8.96 $\pm$ 1.18	9.37 $\pm$ 1.86	.93	.68
Comorbidity					
Present	191(63.67)	90(62.94)	101(64.33)	.42	.28
None	109(36.33)	53(17.67)	56(35.67)		

*M* Mean, *SD* Standard deviation, *EFA* Exploratory factor analysis, *CFA* Confirmatory factor analysis, *HFS* Hip fracture surgery, *THR* Total hip replacement, *BHA* Bipolar hemiarthroplasty, *PFNA* Proximal femoral nail antirotation, *ORIF* Open reduction and internal fixation

^a^Use of aids, walker

#### Stage 7: Evaluation of the scale

### Construct validity

The multivariate normality was assessed by skewness, kurtosis, and Mardia coefficient. The corrected item-total correlation coefficients ranged from 0.128 to 0.615 and the total Cronbach's alpha reliability for the 28 items was 0.82. A total of 24 items were used for EFA, as 4 items with a correlation coefficient less than 0.3 were deleted from the pool.

As the result of primary EFA using PCA as the factor extraction method on 24 items, five factors were extracted. After deleting two items showing the communality of less than 0.5, secondary factor analysis was conducted on the 22 remaining items. There was no problem in communality, but one item was not loaded into any factor and thus was deleted. Tertiary factor analysis was performed on the 21 items. The results showed that the KMO value (0.87) and Bartlett's test of sphericity (χ2=1833.54, *p* <.001) were statistically significant, confirming that the data was suitable for factor analysis. The communality ranged from 0.57 to 0.81, and the factor loadings ranged from 0.51 to 0.87. The cumulative explanatory power of the factors was 66.32% for the 21-item scale.

In EFA, a total of five factors were determined from the data. The first factor included 5 items and was named as ‘functional independence.’ This factor was composed of behaviors to enhance proper nutrition and rehabilitation and focused on functional aspects in the recovery of hip fracture surgery. Factor 2 included five items and was named ‘symptom recognition and managements.’ Factor 3 was composed of five items and was named ‘positive mental health’. Factor 3 included constructs of positive mental health and spiritual support and focused on the older adults’ psychological capacity to adjust themselves to changes in daily life and overcome barriers to rehabilitation. Factor 4 included three items and was named ‘participation in and support for social activity.’ Factor 5 was composed of three items and was named ‘safe environment.’ (Table 2).

**Table 2:** Final results of exploratory factor analysis on HFS-SC (*N=*143)

Factor	Item	Commu-nality	F1	F2	F3	F4	F5
Functional independence	2. I try to do my daily living by myself without any help.	.658	.778				
	8. I take painkiller on prescription after checking the pain intensity.	.573	.727				
	1. I try to be well-nourished.	.632	.706				
	7. I participate in rehabilitation considering my other health issues or the current situation.	.584	.679				
	6. I regularly work out on a daily basis.	.571	.514				
Symptom recognition and management	9. Continuous management is required for hip fracture.	.774		.870			
	11. I know when to visit an emergency room.	.665		.833			
	12. I know what postures or exercises I have to avoid after surgery.	.598		.764			
	22. I regularly visit the clinic to check my medical condition.	.812		.721			
	3. I make sure there is no aggravated pain in the surgical site, no adverse effect, etc. while participating in activity.	.603		.611			
Positive mental health	24. I am careful for not falling again.	.737			.742		
	26. I try to manage my depression from the limitation to move by myself.	.623			.708		
	25. I can deal with stress.	.646			.624		
	28. Religion is very helpful for my recovery.	.634			.579		
	27. Religion helps me think positively.	.608			.563		
Participation in and support for social activities	21. I have a good relationship with family members, friends and neighbors, and often meet with them.	.722				.778	
	20. I have a person who I can ask for help in need.	.660				.723	
	18. I currently participate in economic activity.	.589				.645	
Safe environment	13. I remove any objects that might obstruct the pathway in order not to trip over.	.801					.710
	16. I wear shoes with rubber sole that are easy to put on.	.667					.579
	17. I leave some lights on but not too bright to disturb my sleep.	.652					.563
	Eigen value		5.373	4.076	2.508	2.144	1.816
	Variance(%)		22.387	16.984	10.450	8.933	7.569
	Cumulative Variance(%)		22.837	39.370	49.821	58.753	66.322

CFA was conducted on 157 study participants to confirm the model fit of the five factors derived from the EFA and to verify the convergent and discriminant validity of the constructs. The initial model fit indices tested with 21 items did not meet the standard values (χ2=1182.21 (*p*<.001), χ2/*df*=5.31, RMSEA 0.79, SRMR 0.34, GFI 0.72, TLI 0.74, CFI 0.79). After removing three items with a Standardized regression weights (SRW) less than 0.5 and an Squared multiple correlation (SMC) value of less than 0.5, the secondary CFA was performed on the remaining 18 items. In the analysis of the model of the secondary CFA, the value of χ2 did not fit the standard value (χ2=375.83 (*p*<.001), χ2/*df*=2.14, RMSEA 0.07, SRMR 0.05, GFI 0.88, TLI 0.91, CFI 0.92). However, since χ2 value tends to be easily rejected due to its overt sensitivity to sample size [29], the overall model fit should be evaluated with other fitness indices along with χ2 value. Other goodness-of-fit indices showed that the model was adequate and well explained by the data. Although the GFI value was slightly below the standard of the fit index, the overall model fit was good in consideration of other fit indices such as TLI and CFI (Table 3).

**Table 3:** Confirmatory factor analysis (*N=*157)

Factor	Item	Standaized estimates	SE	Critical Ratio(C.R.)	*p*	AVE	Construct Reliability(CR)
F1	2	.769	.199	12.63	<.001	.758	.926
	8	.792	.165	12.95	<.001		
	1	.803	.275	13.17	<.001		
	6	.737	.129	11.56	<.001		
F2	9	.741	.295	11.82	<.001	.673	.891
	11	.785	.371	12.45	<.001		
	12	.694	.173	11.01	<.001		
	22	.812	.284	13.39	<.001		
F3	24	.733	.372	11.74	<.001	.687	.897
	26	.648	.183	10.31	<.001		
	25	.759	.165	12.21	<.001		
	27	.783	.258	12.97	<.001		
F4	21	.774	.229	12.43	<.001	.715	.882
	20	.692	.216	10.72	<.001		
	18	.638	.147	10.11	<.001		
F5	13	.693	.196	10.94	<.001	.763	.906
	16	.731	.134	11.32	<.001		
	17	.617	.103	9.85	<.001		
Fitness index	χ^2^(p)	χ^2^/df	RMSEA	SRMR	GFI	TLI	CFI
Criteria	(.05)	3	.10	.08	.90	.90	.90
Model	375.83(<.001)	2.14	.07(.06~.10)	.05	.88	.91	.92

*AVE* Average variance extracted, *F1* Functional independence, *F2* Symptom recognition and management, *F3* Positive mental health, *F4* Participation in and support for social activities, *F5* Safe environment

**Fig. 2:**
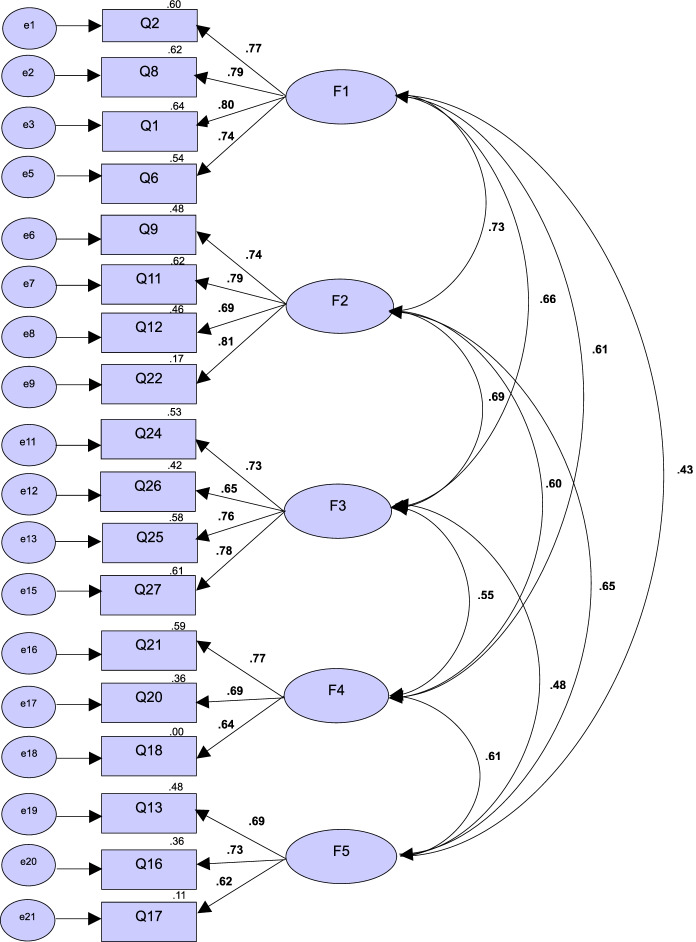
Final model of self-care scale for older adults undergoing

In terms of convergent validity, all ranges satisfied the recommended criteria of factor loadings, C.R ($>\pm$ 1.96), AVE ($>$ 0.5), and CR ($>$ 0.7), supporting the convergent validity of the scale. Discriminant validity was evaluated by examining if the AVE value is greater than the value of squared correlation coefficients [35, 37]. In this study, as shown in Table 3, the discriminant validity was secured as all of the AVE values were larger than the values of squared correlation coefficients among subdomains (Table 3, Fig 2).

Concurrent validity was examined with the Pearson correlation coefficients between the HFS-SC scale and the EQ-5D-5L. There were significant positive correlations among the subdomains of the HFS-SC and the EQ-5D-5L. There was also a high correlation between the HFS-SC and the EQ-5D-5L total scores (*r* = 0.74, *p*<.001). There were moderate correlations between subdomains of the HFS-SC and the EQ-5D-5L (*r* = 0.66, *p*<.001 for factor 1; *r* = 0.63, *p*<.001for factor 2; *r* = 0.54, *p*<.001 for factor 3; *r* = 0.50, *p*<.001 for factor 4; and *r* = 0.68, *p*<.001 for factor 5), supporting the concurrent validity of the HFS-SC.

### Reliability

To examine test-retest reliability, 52 subjects were evaluated using the HFS-SC scale at four weeks after the baseline measurement point. The ICC of the HFS-SC scores at the two time points was 0.82. In internal consistency reliability, Cronbach's alpha was 0.91 for the HFS-SC. By subdomain, Cronbach's alpha reliabilities were 0.88 for factor 1, 0.89 for factor 2, 0.86 for factor 3, 0.83 for factor 4, and 0.85 for factor 5.

#### Stage 8: Scale optimization

The psychometric properties of the HFS-SC were examined and confirmed. The HFS-SC consists of 18 items. The scale is composed of 5 sub-domains: functional independence, symptom recognition and management, positive mental health, participation and support in social activities, and safe environment. The total score ranges from 18 to 90, with a higher score indicating a higher level of self-care for older adults who underwent HF surgery.

## Discussion

Treatment of older adults who undergo HF surgery is aimed at improved quality of life through recovery of function and mobility [51]. The importance of accurate and specific assessment of perceived self-care ability cannot be overemphasized [52], and it is necessary to develop a specific scale that can measure self-care levels in patients with HF [18]. In view of the need to focus on healing in addition to outcomes of health status after HF surgery, the Hip Fracture Surgery Self-Care Scale (HFS-SC) was developed for multidimensional evaluation of self-care and is specifically tailored to patients with HF.

In this study, several indicators were used to confirm the validity of the HFS-SC, which contains five subdomains of the 18-item scale. The five essential attributes of self-care in older adults who undergo HF surgery include functional independence, recognition and management of symptoms, positive mentality, participation in and support for social activities, and a safe environment. In essence, optimal self-care in older adults who undergo HF surgery should include maximization of physical function, awareness of one’s health status, engagement with ADLs with a positive attitude, maintenance of a social support system with active social participation, and efforts directed toward a safe living environment. Multidimensionality of the HFS-SC was supported by CFA, which showed a model fit, convergent and discriminant validity of the scale, and high correlation of items in each subdomain. The HFS-SC was also significantly correlated with the EQ-5D-5L, which confirms the concurrent validity of the scale. This finding concurs with the view that quality of life in older adults with HF was negatively affected in the absence of appropriate self-care, when measured using the EQ-5D scale [7].

HFS-SC stability was established using test-retest reliability. The internal consistency reliability of the scale was also confirmed (the Cronbach’s alpha value of the total scale was 0.91). The internal consistency reliability of the HFS-SC was equivalent or higher than that of a scale used to measure self-care in older adults with hypertension (0.92) [53] or that of a scale used to measure self-care in older adults with dysphasia (0.90) [54]. Therefore, the HFS-SC is considered an accurate tool to assess self-care in older adults who undergo HF surgery.

The HFS-SC differs from other scales because it indicates both the characteristics of the HF per se and the transition of patients with HF from the acute stage of treatment to routine life and activities at home after discharge. Inclusion of physical, cognitive, emotional, social, and environmental domains in the HFS-SC ensures multidimensionality to accurately estimate a patient’s functional abilities at home based on the disease experience, disease characteristics, and perceived health status.

From a theoretical standpoint, our study expands the scope of Orem's self-care theory [10] by applying the theory in older patients who underwent HF surgery. Orem’s self-care theory is predominantly used for patients with cancer [55, 56] or chronic diseases [57–59] and is rarely used in a comprehensive manner to assess recovery from a specific clinical condition. The application of the HFS-SC is most effective after completion of acute rehabilitation and return to routine ADLs at home in older adults who undergo HF. Based on self-care levels measured using the HF-SC, healthcare providers can evaluate the functional performance of older adults who undergo HF surgery and psychological adjustments in patients during the post-fracture period in the community or in outpatient departments.

HFS-SC scores may serve as a useful guideline for nurses to develop interventions to improve patient monitoring and eventually help to improve quality of life. Further research is warranted to investigate the psychometric properties of the HFS-SC through replication studies and to determine the usefulness of the HFS-SC for estimation of health outcomes, such as medical expenses, the psychological burden on the patient’s family, and mortality.

Following are the limitations of this study: Data were obtained only from a few regions across Korea; therefore, the generalizability of our findings is questionable. The concept of self-care may vary across cultures; therefore, the content validity and cultural sensitivity of the scale items should be investigated in a greater number of geographical areas. Some behaviors, such as confirmation of pain intensity may be difficult in the oldest old age group; therefore, future studies should investigate the psychometric properties of the scale across various age groups. The factor structure of the HFS-SC should also be confirmed in future replication studies to validate the findings of our study.

## Conclusion

The HFS-SC, a new scale to measure the level of self-care in older adults who underwent HF surgery, was developed, and its psychometric properties were examined. The HFS-SC consists of 18 items and is relatively easy to apply to older adults.

## Supplementary Information

**Additional file 1: Appendix A.** The HFS-SC scale.

## Data Availability

The datasets generated and/or analyzed during the current study are not publicly available due to privacy or ethical restrictions but are available from the corresponding author on reasonable request.

## Abbreviations

ADL: Activities of daily living
AVE: Average variance extracted
CFA: Confirmatory factor analysis
CFI: Comparative fit index\
CR: Construct reliability
C.R: Critical Ratio
EFA: Exploratory factor analysis
EQ-5D-5L: EuroQol-5dimensions 5Levels
GFI: Goodness of fit index
HF: Hip fracture
HFS-SC: Hip fracture surgery self-care
IADL: Independence in the activities of daily living
ICC: Intra-class correlation coefficient
I-CVI: Item-level content validity index
M: Mean
MLE: Maximum likelihood estimator
RMSEA: Root mean square error of approximation
S-CVI/AVE: Scale-level content validity index/Average
SD: Standard deviation
SRMR: Standardized root mean square residual
TLI: Turker-lewis index
