# Supplementary material for: Development and validation of a self-care scale for older adults undergoing hip fracture surgery: the HFS-SC
Source: BMC Nurs. 2022 Jul 22;21:197. doi: 10.1186/s12912-022-00982-3 (PMC9308275; doi:10.1186/s12912-022-00982-3)
Supplement: Supplementary file 1 — Additional file 1: Appendix A. The HFS-SC scale. [file 12912_2022_982_MOESM1_ESM.docx]

Appendix A. The HFS-SC scale

| Item | Strongly disagree | Disagree | Neutral | Agree | Strongly agree |
| --- | --- | --- | --- | --- | --- |
| 1. I try to do my daily living by myself without any help. |  |  |  |  |  |
| 2. I take painkiller on prescription after checking the pain intensity. |  |  |  |  |  |
| 3. I try to be well-nourished. |  |  |  |  |  |
| 4. I regularly work out on a daily basis. |  |  |  |  |  |
| 5. Continuous management is required for hip fracture. |  |  |  |  |  |
| 6. I know when to visit an emergency room. |  |  |  |  |  |
| 7. I know what postures or exercises I have to avoid after surgery. |  |  |  |  |  |
| 8. I regularly visit the clinic to check my medical condition. |  |  |  |  |  |
| 9. I am careful for not falling again. |  |  |  |  |  |
| 10. I try to manage my depression from the limitation to move by myself. |  |  |  |  |  |
| 11. I can deal with stress |  |  |  |  |  |
| 12. Religion helps me think positively. |  |  |  |  |  |
| 13. I have a good relationship with family members, friends and neighbors, and often meet with them. |  |  |  |  |  |
| 14. I have a person who I can ask for help in need. |  |  |  |  |  |
| 15. I currently participate in economic activity. |  |  |  |  |  |
| 16. I remove any objects that might obstruct the pathway in order not to trip over. |  |  |  |  |  |
| 17. I wear shoes with rubber sole that are easy to put on. |  |  |  |  |  |
| 18. I leave some lights on but not too bright to disturb my sleep. |  |  |  |  |  |
